# Distresses reported by physicians and nurses toward peculiarities of patients with head and neck cancer at a university general hospital in Brazil: A qualitative study

**DOI:** 10.1192/j.eurpsy.2021.1157

**Published:** 2021-08-13

**Authors:** E. Turato, A.C. Bispo, J.R. Rodrigues, C.S. Lima

**Affiliations:** Medical Psychology And Psychiatry, University of Campinas, Campinas, Brazil

**Keywords:** distress, Qualitative Research, medical psychology, head neck cancer

## Abstract

**Introduction:**

Contextualization: health professionals’ anguish towards the patient with head and neck cancer (HNC) permeates clinical issues: the location of the tumour, if advanced diagnosis, the psychosocial features of the patient. The perception, coming from patients as undesirable, refers to the conflict of how to deal with one’s own anguishes.

**Objectives:**

AIM: To explore and interpret how anguish experienced by physicians and nurses are mobilized regarding to the clinical and psychosocial peculiarities of patients with HNC.

**Methods:**

Strategies: Clinical-qualitative design; semi-directed interview with open-ended questions in depth. Trigger question: “Tell me about the management of the patient with …”. Ten interviewees (06 nurses and 04 resident doctors) from a university oncology outpatient. Intentional sample. Clinical-Qualitative Content Analysis with psychodynamic concepts. Findings validated by peers at the Laboratory of Clinical-Qualitative Research at the University of Campinas, Brazil.

**Results:**

Topics: the treatment of the speeches resulted in three emerging categories: (1) Cancer is literally on the face: self-perception of peculiarities; (2) An appalling illness: dealing with the ‘deteriorated’; (3) To naturalize without trivializing: handling with their own anguish.

**Conclusions:**

Final considerations: The anguish of health professionals who deals with the HNC patient consists of the feelings, which are not exposed, because they are not organized and neither understood as natural feelings. It is up to them to seek neutrality to minimize the anguish present in the conflict of not manifesting thoughts considered inadequate by the patient, avoiding moral judgments and conflicts. Balint groups are recommended to attend emotional demands of health professionals.

